# The Effects of Modified Simiao Decoction in the Treatment of Gouty Arthritis: A Systematic Review and Meta-Analysis

**DOI:** 10.1155/2017/6037037

**Published:** 2017-03-08

**Authors:** Ya-Fei Liu, Ying Huang, Cai-Yu-Zhu Wen, Jun-Jun Zhang, Guo-Lan Xing, Sheng-Hao Tu, Zhe Chen

**Affiliations:** ^1^Department of Nephrology, The First Affiliated Hospital of Zhengzhou University, 1 Jianshe East Road, Zhengzhou, Henan 450052, China; ^2^Institute of Integrated Traditional Chinese and Western Medicine, Tongji Hospital, Tongji Medical College, Huazhong University of Science and Technology, 1095 Jiefang Avenue, Wuhan, Hubei 430030, China; ^3^Hubei University of Chinese Medicine, 1 Huangjiahu West Road, Wuhan, Hubei 430065, China

## Abstract

The modified Simiao decoctions (MSD) have been wildly applied in the treatment of gouty arthritis in China. However, the evidence needs to be evaluated by a systematic review and meta-analysis. After filtering, twenty-four randomised, controlled trials (RCTs) comparing the effects of MSD and anti-inflammation medications and/or urate-lowering therapies in patients with gouty arthritis were included. In comparison with anti-inflammation medications, urate-lowering therapies, or coadministration of anti-inflammation medications and urate-lowering therapies, MSD monotherapy significantly lowered serum uric acid (*p* < 0.00001, mean difference = −90.62, and 95% CI [−128.38, −52.86]; *p* < 0.00001, mean difference = −91.43, and 95% CI [−122.38, −60.49]; *p* = 0.02, mean difference = −40.30, and 95% CI [−74.24, −6.36], resp.). Compared with anti-inflammation medications and/or urate-lowering therapies, MSD monotherapy significantly decreased ESR (*p* < 0.00001; mean difference = −8.11; 95% CI [−12.53, −3.69]) and CRP (*p* = 0.03; mean difference = −3.21; 95% CI [−6.07, −0.36]). Additionally, the adverse effects (AEs) of MSD were fewer (*p* < 0.00001; OR = 0.08; 95% CI [0.05, 0.16]). MSD are effective in the treatment of gouty arthritis through anti-inflammation and lowering urate. However, the efficacy of MSD should be estimated with more RCTs.

## 1. Introduction

Gouty arthritis, one of the most common forms of inflammatory arthritis, is characterized by hyperuricemia and deposition of monosodium urate. The prevalence of gout among US men in 2007-2008 was 5.9%, and the prevalence among women was 2.0% [1]. Global epidemiology of gout indicated that the distribution of gout was uneven across the world, with prevalence being highest in Pacific countries. Developed countries incline to have a higher load of gout than developing countries, and the prevalence and incidence seem to be increasing [2].

Conventional therapies for gouty arthritis include anti-inflammation medications (colchicine, nonsteroidal anti-inflammatory drugs, and glucocorticoids) and urate-lowering therapies (allopurinol, benzbromarone, and febuxostat). However, allopurinol could lead to severe hypersensitivity and was limited in patients with renal insufficiency [3, 4]. The Food and Drug Administration implemented enforcement action against companies illegally marketing unapproved single-ingredient oral colchicine due to its toxicity [5]. Meanwhile, the relatively high medical care cost of febuxostat restricts its application in the developing countries. Therefore, it is imperative to explore new available approaches for gouty arthritis, especially complementary and alternative medicine.

Plant-based medicines are widely employed in the treatment of gouty arthritis in China for thousands of years. In Chinese medicine, gouty arthritis is correlated with dampness, heat, sputum, and stasis. Among numerous effective prescriptions, Simiao pill, derived from Ermiao powder, and described in a famous traditional Chinese medicine monograph Chengfang Biandu in Qing Dynasty of China, was wildly applied for treatment of gouty arthritis. It is composed of four individual herbs:* Rhizoma Atractylodis*,* Cortex Phellodendri*,* Radix Achyranthis Bidentatae*, and* Semen Coicis*. Moreover, Simiao pill and its derivative prescriptions showed their beneficial efficacy in treating gouty arthritis and hyperuricemia in vitro and in vivo [6–9]. To cope with the intricate pathologic states of gouty arthritis in different stages, different modified Simiao decoctions (MSD) have been developed based on different syndromes and traditional Chinese medicine theory. Our previous studies also demonstrated that MSD and their major component berberine were of use in attenuating the monosodium urate crystals-induced inflammation [9, 10].

While MSD have been most frequently used for a long time in treating gouty arthritis, there exist a series of issues. In this regard, most of the clinical researches have arisen from uncontrolled clinical studies or from retrospective reports, and few multicentre clinical trials have been conducted to validate the effects of MSD in the treatment of gouty arthritis. In addition, the scientific evidence that MSD are as effective as other conventional treatments in treating gouty arthritis remains to be confirmed further. In regard to safety concerns, the safety of long-term MSD intake for gouty arthritis is uncertain. Given these uncertainties, it is essential to evaluate the pertinent studies to systematically review the potential effects and safety of the long-term intake of MSD in the treatment of gouty arthritis.

## 2. Materials and Methods

To confirm the accuracy of our systemic review and meta-analysis, we designed and performed our results applying a checklist of items that were as consistent as possible with the Preferred Reporting Items for Systemic Review and Meta-Analyses (PRISMA) statement [11].

### 2.1. Search Strategy

We searched the following foreign databases to identify trials: PubMed, the Cochrane Library, and Clinical Trials.gov. Meanwhile, we retrieved Chinese databases, such as the CNKI Database, WanFang Database, and Chinese Clinical Trial Register. All of the databases were searched from their available dates of inception to the latest issue (September 2016).

Different search strategies were combined as follows. For the English databases, we used free text terms, such as “simiao” and “gouty arthritis” or “gout”. For the Chinese databases, free text terms were applied, such as “simiao” and “Tong Feng” (which means gout in Chinese). A filter for clinical studies was applied. To collect sufficient trials, the reference lists of relevant articles were also searched to identify additional studies.

### 2.2. Selection Criteria

The randomised controlled trials (RCTs) were included regardless of blinding, publication status, or language. Studies were included for analysis if they satisfied the following criteria: (1) For the types of interventions, treatments with MSD alone in RCTs were considered. The control groups consisted of treatments with anti-inflammation medications and/or urate-lowering therapies. (2) The study was an RCT with a parallel or crossover design. And (3) Patients were enrolled who were diagnosed as having gouty arthritis, according to the classified criteria for gouty arthritis by American Society of Rheumatism in 1997 or Chinese diagnostic criteria for gouty arthritis.

In this review, studies using any MSD combined with western medicine or drugs for external use or acupuncture were excluded. We also excluded case reports, reviews, retrospective studies, or studies without scheduled outcomes. For obviously repeated studies, the authors of the studies were contacted to clarify any ambiguities. If the authors could not be connected, the first published study was considered to be the original. Studies were also excluded if the control groups were not conventional therapies for gouty arthritis. RCTs were also eliminated from our analysis that lacked sufficient data to allow for calculating the mean changes from the baseline to the endpoints. Two reviewers selected the articles independently. We generated a flow diagram of the study selection in accordance with the PRISMA requirements.

### 2.3. Data Extraction and Management

The data were extracted by two independent reviewers, and any divergences were resolved by consensus or were arbitrated by a third reviewer. The studies' quality was assessed according to Cochrane handbook 5.3. The risk of bias included the following items: (A) random sequence generation (selection bias); (B) allocation concealment (selection bias); (C) blinding of participants and personnel (performance bias); (D) blinding of outcome assessment (detection bias); (E) incomplete outcome data (attrition bias); (F) selective reporting (reporting bias); (G) other bias.

The primary outcome was serum uric acid (SUA). The secondary outcomes consisted of erythrocyte sedimentation rate (ESR), C-reactive protein (CRP), and white blood cell (WBC). AEs were also collected from the studies. For the trials that applied a three-armed group design, the outcomes of the groups were extracted if they met the inclusion criteria. In cases in which the outcomes were vague or absent in the articles, we endeavoured to contact the authors. If the authors were not connected, we extracted the data by consensus.

### 2.4. Data Synthesis and Analysis

The effects of MSD intake on patients with gouty arthritis were calculated as differences between the MSD groups and the control groups, employing Review Manager meta-analysis software, version 5.3. To ensure the credibility of the results, the net changes in all of the outcomes were calculated as the mean differences (MSD minus control) in changes (endpoint minus baseline) for parallel trials. We calculated weighted mean differences and 95% confidence intervals (CIs) for continuous data. Heterogeneity was evaluated via the chi-square test and Higgins *I*^2^ test. A fixed-effect model was employed when the studies in the group were sufficiently alike (*p* > 0.10); otherwise, a random-effects model was applied. A *Z* score was calculated to determine the overall effect, with significance set at *p* < 0.05. Publication bias was detected by Egger's regression asymmetry test and Begg's test when the number of included trials ≥ 5 (Stata software, version 12.0).

To minimise the clinical heterogeneity, in terms of SUA, we performed three subgroup analyses: MSD compared with anti-inflammation medications; MSD compared with urate-lowering therapies; and cointervention of anti-inflammation medications and urate-lowering therapies compared with MSD alone.

## 3. Results

### 3.1. Study Selection

**Figure 1:**
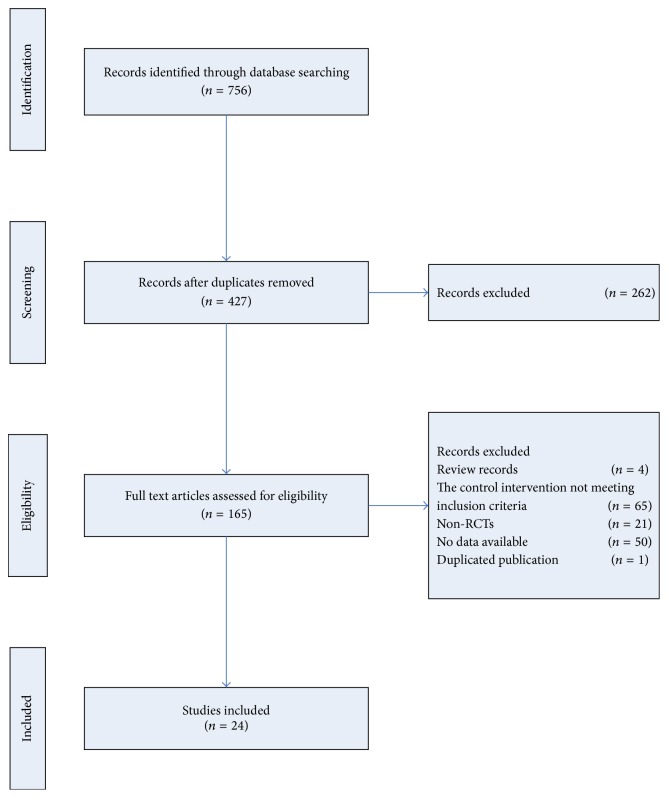
Study selection flow chart.

The process of study selection was indicated in Figure 1. According to the prespecified selection criteria defined in the Materials and Methods, 24 RCTs were included in the meta-analysis. In the anti-inflammation medications subgroup, we searched ten studies [13–16, 18, 20, 22, 24, 26, 33]. One study compared oral MSD and/or external use with colchicine and allopurinol, and oral MSD group was selected in comparison with colchicine and allopurinol [31]. The trial of Shi et al. compared three different MSD with indomethacin and benzbromarone, and we just extracted the data of Group III and control group [6]. In the urate-lowering therapies group, we searched three studies [7, 17, 23]. In the combined therapy subgroup, we searched eleven studies [6, 12, 19, 21, 25, 27–32]. The characteristics of the studies were summarised in Table 1. Together, those studies included a total of 1895 participants.

### 3.2. Study Descriptions

The included RCTs were published as full text between 2006 and 2016. The duration of intervention in the included trials ranged from 3 days to 30 days. All of the trials were originated from China. Two studies were published in English [6, 7], while 22 studies were published in Chinese. All of the RCTs were conducted as single-centre trials. One trial was a master's degree thesis [14], and the others were journal articles. All of the trials were performed in mainland China. The important sources and compositions of MSD were indicated in Table 2.

### 3.3. Quality of the Included Studies

As indicated in Figure 3, most of the included trials were of low quality due to unclear randomisation, deficient allocation concealment, inadequate blinding, and undescribed withdrawals and dropouts, compared with two trials [6, 21] that were of moderate quality. Meanwhile, high risk of other bias could exist in three trials [22, 25, 27].

### 3.4. Publication Bias

**Figure 2:**
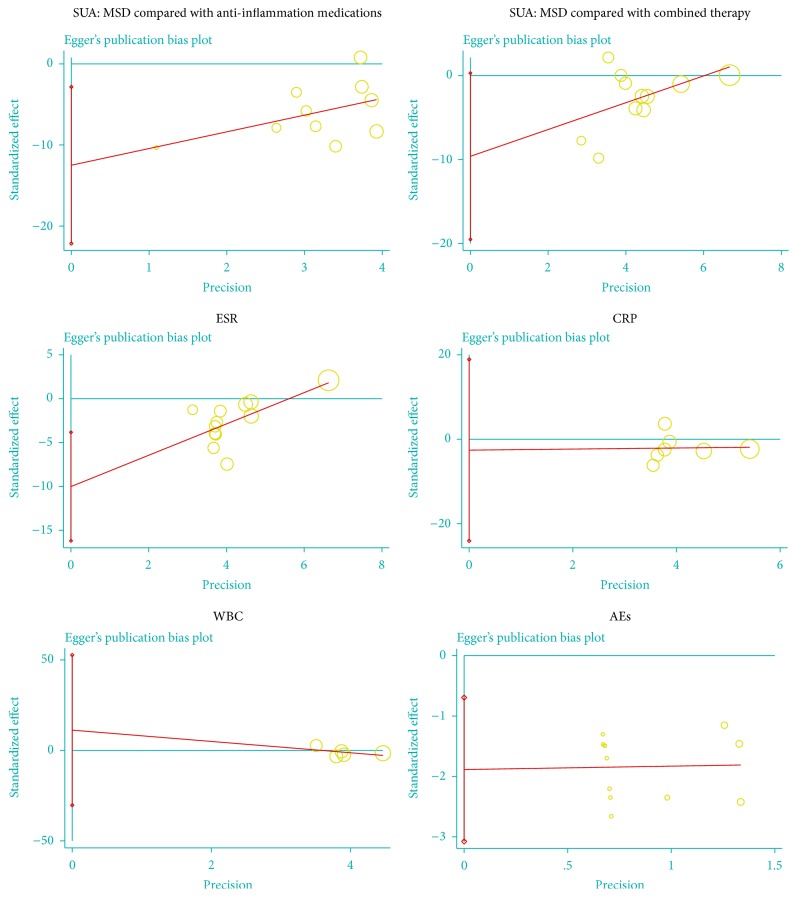
Publication bias in the included trials. Egger's linear regression test for detecting publication bias. Note: MSD: modified Simiao decoction; SUA: serum uric acid; ESR: erythrocyte sedimentation rate; CRP: C-reactive protein; WBC: white blood cell; AEs: adverse effects. “О” is a size graph symbol for the weight of each included study. The distance between two diamonds on the second vertical bar on the left represents the 95% CI for the intercept.

Egger's publication bias plots and Begg's test displayed that there were significant publication biases for three outcomes in terms of SUA (when compared with anti-inflammation medications), ESR, and AEs. Meanwhile, there were no publication biases for three outcomes in terms of SUA (with cointervention of anti-inflammation medications and urate-lowering therapies), CRP, and WBC. As presented in Figure 2, the calculated *p* values exceeded 0.05 for the three outcomes among the studies (SUA, *p* = 0.056; CRP, *p* = 0.771; WBC, *p* = 0.453), and the 95% CI for the intercept included zero. However, these results cannot be regarded as convincing except SUA (with cointervention of anti-inflammation medications and urate-lowering therapies), because there were fewer than ten trials.

### 3.5. Effects of Interventions

#### 3.5.1. Effects of MSD on SUA

**Figure 3:**
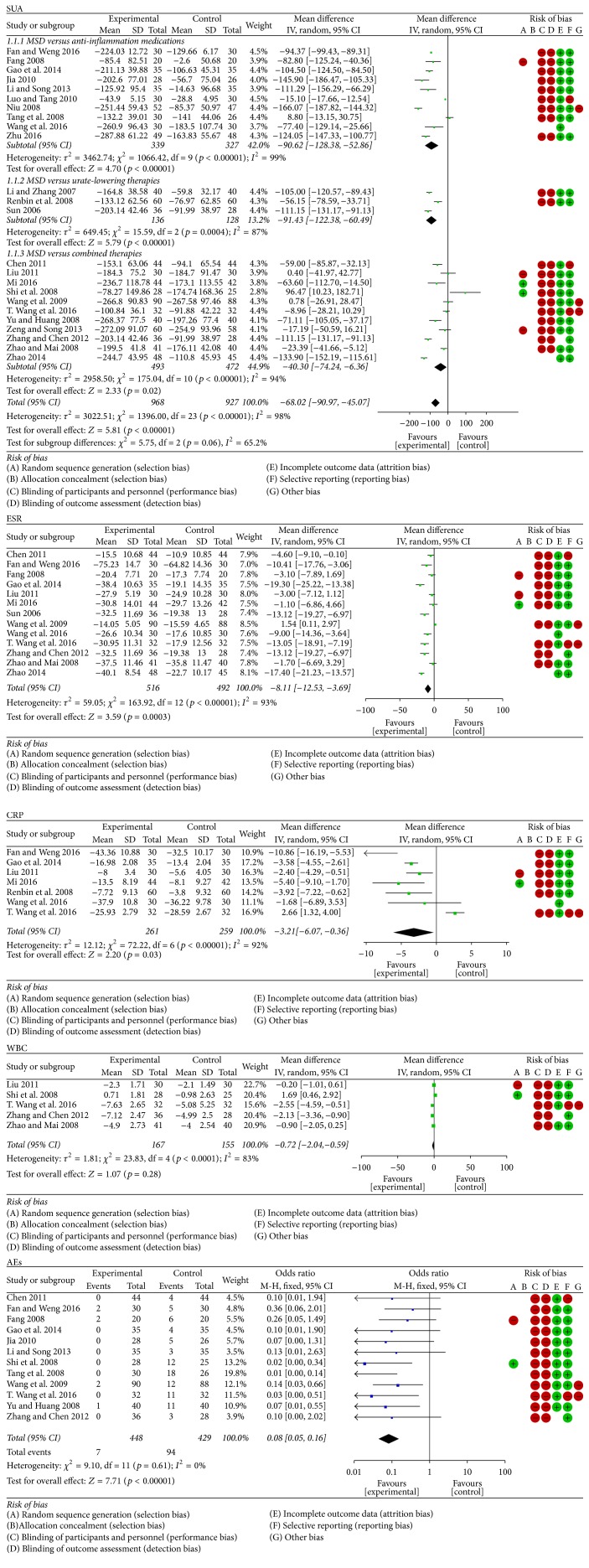
Forest plots for the comparison of the effects of MSD and conventional therapies and risk of bias. Note: MSD: modified Simiao decoction; SUA: serum uric acid; ESR: erythrocyte sedimentation rate; CRP: C-reactive protein; WBC: white blood cell; AEs: adverse effects.

*(1) MSD Compared with Anti-Inflammation Medications*. Ten trials (involving 666 patients) compared the therapeutic effects of MSD and anti-inflammation medications [13–16, 18, 20, 22, 24, 26, 33]. The number of trial participants ranged from 20 to 52, with the trial duration varying from 3 days to 28 days. As illustrated in Figure 3, there was statistical heterogeneity between the studies. The MSD groups were superior to the anti-inflammation medication groups in terms of lowering the SUA (*p* < 0.00001; mean difference = −90.62; 95% CI [−128.38, −52.86]).

*(2) MSD Compared with Urate-Lowering Therapies*. Three trials (involving 264 patients) compared the therapeutic effects of MSD and urate-lowering therapies [7, 17, 23]. The number of trial participants ranged from 28 to 60, with the trial duration varying from 14 days to 30 days. As illustrated in Figure 3, there was statistical heterogeneity between the studies. The MSD groups were superior to the urate-lowering therapies groups with regard to lowering the SUA (*p* < 0.00001; mean difference = −91.43; 95% CI [−122.38, −60.49]).

*(3) MSD Compared with Combined Therapies*. Eleven trials (involving 965 patients) compared the therapeutic effects of MSD and combined therapies [6, 12, 19, 21, 25, 27–32]. The number of trial participants ranged from 25 to 90, with the trial duration varying from 7 days to 30 days. As illustrated in Figure 3, there was statistical heterogeneity between the studies. The MSD groups were superior to the combined therapies groups regarding lowering the SUA (*p* = 0.02; mean difference = −40.30; 95% CI [−74.24, −6.36]).

#### 3.5.2. Effects of MSD on ESR

ESR was reported in thirteen trials (involving 1008 patients) [12–15, 19, 21, 23, 25–27, 30–32]. The number of trial participants ranged from 20 to 90, with the trial duration varying from 5 days to 30 days. As illustrated in Figure 3, there was statistical heterogeneity between the studies. The MSD groups were superior to the control groups regarding decreasing the ESR (*p* < 0.00001; mean difference = −8.11; 95% CI [−12.53, −3.69]).

#### 3.5.3. Effects of MSD on CRP

CRP was determined in seven trials (involving 520 patients) [7, 13, 15, 19, 21, 26, 27]. The number of trial participants ranged from 30 to 60, with the trial duration varying from 7 days to 28 days. As illustrated in Figure 3, there was statistical heterogeneity between the studies. The MSD groups were superior to the control groups regarding reducing the CRP (*p* = 0.03; mean difference = −3.21; 95% CI [−6.07, −0.36]).

#### 3.5.4. Effects of MSD on WBC

WBC was detected in five trials (involving 322 patients) [6, 19, 27, 30, 31]. The number of trial participants ranged from 25 to 41, with the trial duration varying from 7 days to 30 days. As illustrated in Figure 3, there was statistical heterogeneity between the studies. There was no significant reduction in terms of WBC between MSD and control groups (*p* = 0.28; mean difference = −0.72; 95% CI [−2.04, 0.59]).

### 3.6. The AEs of MSD

AEs were reported in twelve trials (involving 111 patients) [6, 12–16, 18, 24, 25, 27, 28, 30]. The number of cases ranged from 0 to 18. As illustrated in Figure 3, there was no statistical heterogeneity between the studies. The MSD groups were superior to the control groups regarding the AEs (*p* < 0.00001; OR = 0.08; 95% CI [0.05, 0.16]).

## 4. Discussion

Although several systematic reviews and meta-analyses concerning the efficacy of MSD in the treatment of gouty arthritis have been performed, the primary outcome of these systematic reviews was clinical response rate which was judged by Chinese criteria [34–37]. Except for the clinical response rate, the meta-analysis published by Xie (8 trials with 633 participants) also narrated the chemical profiles, such as SUA, ESR, and CRP [37]. Meanwhile, Xie divided the studies into two subgroups (with or without urate-lowering therapies) when analysing the effects of MSD on SUA [37]. The two systematic reviews published by Du et al. only reported the clinical response rate and safety [35, 36]. Unlike the previous reviews, we included 24 trials and set three subgroups when determining the effects of MSD on SUA. However, the clinical response rate was not detected because it was easily influenced by subjective factors. Furthermore, we added more new trials published after the previous reviews. Thus, our systemic review is different from the previous reviews.

In terms of SUA, our results were consistent with Xie and Zhou et al. [34, 37]. These showed that MSD were of use in lowering SUA and they could be applied in the treatment of hyperuricemia and reduce the incidence of gouty arthritis.

Many of our results were in line with the results of Xie [37] between the MSD-treated groups and the control groups in terms of ESR and CRP. These indicated that MSD played an important role in anti-inflammation and decreasing disease activity of gouty arthritis, which further validated the effects of MSD in the treatment of gouty arthritis.

Unlike the previous review by Xie [37], we conducted three subgroups when comparing the effects of MSD and control groups on SUA. In addition, the WBC was observed in our systemic review. The results demonstrated that MSD had no beneficial effects in decreasing WBC, which was due to the aseptic inflammation of gouty arthritis.

Our results illustrated that MSD surpassed control groups with regard to AEs, which were in harmony with Xie and Du et al. [35–37]. The most common AEs with MSD were diarrhea, nausea, and vomit, and they could be alleviated with or without dose reductions. These showed that MSD were safe in the treatment of gouty arthritis.

However, several limitations of this meta-analysis should be noted. First, all of the included trials were performed in Chinese populations, which implied high risk of selection bias. This fact could have influenced the applicability of MSD to populations of other countries. Second, most of the studies published in Chinese were of poor quality concerning their designs, reporting, and methodologies. Third, the heterogeneity between the trials included in each subgroup was also significant. We believe that differences in control groups, components of MSD, doses, and durations of treatment were responsible for the heterogeneity. Fourth, the control groups, including anti-inflammation medications and/or urate-lowering therapies, were different. Hence, it is unsuitable to compare the effects of MSD and the control groups. Taking these facts into account, we should carefully interpret all of the conclusions due to the substantial methodological and clinical variety of the trials.

## 5. Conclusions

In summary, MSD monotherapy is superior to anti-inflammation medications and/or urate-lowering therapies in the treatment of gouty arthritis. Meanwhile, the AEs of MSD were mild. Based on their bioactivity, MSD function as anti-inflammation as well as lowering uric acid. Considering the low methodological quality of the included trials, more large and well-designed RCTs are needed before we can recommend MSD to replace western medicine.

## Figures and Tables

**Table 1:** Clinical and demographic characteristics of the patients with gouty arthritis.

Study (ref.)	Number of participants		Age (years)		Intervention			Outcomes
	Experimental	Control	Experimental	Control	Experimental	Control	Duration (days)	
Chen 2011 [12]	44	44	48.5 ± 9.26	49.01 ± 8.95	MSD	Colchicine; allopurinol	14	SUA; ESR; AEs
Fan and Weng 2016 [13]	30	30	48.0 ± 3.8	46.0 ± 3.6	MSD	Meloxicam	28	SUA; ESR; CRP; AEs
Fang 2008 [14]	20	20	57.35 ± 14.54	53.05 ± 14.47	MSD	Loxoprofen sodium	5	SUA; ESR; AEs
Gao et al. 2014 [15]	35	35	44.2 ± 7.2	39.4 ± 8.1	MSD	Colchicine; Etocoxib	7	SUA; ESR; CRP; AEs
Jia 2010 [16]	28	26	43.0 ± 10.8	44.0 ± 11.3	MSD	Colchicine	14	SUA; AEs
Li and Zhang 2007 [17]	40	40	47.5 ± 3.17	46.8 ± 2.74	MSD	Allopurinol	14	SUA
Li and Song 2013 [18]	35	35	NR	NR	MSD	Diclofenac sodium	7	SUA; AEs
Liu 2011 [19]	30	30	NR	NR	MSD	Allopurinol; nimesulide	21	SUA; ESR; CRP; WBC
Luo and Tang 2010 [20]	30	30	NR	NR	MSD	Colchicine; diclofenac sodium	3	SUA
Mi 2016 [21]	44	42	46.12 ± 8.21	45.42 ± 7.85	MSD	Allopurinol; nimesulide	21	SUA; ESR; CRP
Niu 2008 [22]	52	47	NR	NR	MSD	Colchicine	7	SUA
Renbin et al. 2008 [7]	60	60	46.9 ± 3.37	46.8 ± 3.24	MSD	Allopurinol	30	SUA; CRP
Shi et al. 2008 [6]	28	25	54.0 ± 12.5	52.9 ± 13.2	MSD	Indomethacin; benzbromarone	14	SUA; WBC; AEs
Sun 2006 [23]	36	28	53.2 ± 7.8	54.0 ± 8.3	MSD	Benzbromarone	28	SUA; ESR
Tang et al. 2008 [24]	30	26	NR	NR	MSD	Colchicine	15	SUA; AEs
Wang et al. 2009 [25]	90	88	NR	NR	MSD	Colchicine; benzbromarone	14	SUA; ESR; WBC; AEs
Wang et al. 2016 [26]	30	30	36.1 ± 2.8	36.7 ± 2.5	MSD	Colchicine	14	SUA; ESR; CRP
Wang et al. 2016 [27]	32	32	NR	NR	MSD	Allopurinol; NSAIDs	10	SUA; ESR; CRP; WBC; AEs
Yu and Huang 2008 [28]	40	40	48.2 ± 8.6	46.0 ± 9.7	MSD	Nimesulide; allopurinol	14	SUA; AEs
Zeng and Song 2013 [29]	60	58	NR	NR	MSD	Colchicine; allopurinol	14	SUA
Zhang and Chen 2012 [30]	36	28	45.2 ± 7.8	43.4 ± 8.3	MSD	Colchicine; allopurinol; Voltaren or celecoxib	7	SUA; ESR; WBC; AEs
Zhao and Mai 2008 [31]	41	40	NR	NR	MSD	Colchicine; allopurinol	30	SUA; ESR; WBC; AEs
Zhao 2014 [32]	48	45	NR	NR	MSD	Colchicine; allopurinol; Voltaren	7	SUA; ESR
Zhu 2016 [33]	49	48	45.77 ± 7.08	45.45 ± 7.18	MSD	Colchicine	15	SUA

Note: MSD: modified Simiao decoction; SUA: serum uric acid; ESR: erythrocyte sedimentation rate; CRP: C-reactive protein; WBC: white blood cell; AEs: adverse effects; NR, no report. Values are mean ± standard deviation (SD).

**Table 2:** The important sources and compositions of MSD.

Studies	Components of MSD
Chen 2011 [12]	*Rhizoma Atractylodis Macrocephalae, Rhizoma Atractylodis, Radix Angelicae Sinensis, Radix Saposhnikoviae, Radix Glycyrrhizae, Radix Puerariae, Radix Achyranthis Bidentatae, Radix Sophorae Flavescentis, Radix Scutellariae, Cortex Phellodendri, Radix Ginseng, Rhizoma Cimicifugae, Semen Coicis, Rhizoma Et Radix Notopterygii, Virgate Wormwood Herb, Anemarrhena asphodeloides *Bunge*, Polyporus umbellatus, Alisma orientalis*

Fan and Weng 2016	*Caulis Lonicerae, Caulis Trachelospermi, Gymnema alternifolium, Caulis Sinomenii, Smilax bockii *Warb*, Spatholobus suberectus, Cortex Phellodendri, Radix Achyranthis Bidentatae, Semen Coicis, Rhizoma Atractylodis*

Fang 2008	*Cortex Phellodendri, Radix Achyranthis Bidentatae, Semen Coicis, Rhizoma Atractylodis*

Gao et al. 2014	*Cortex Phellodendri, Radix Achyranthis Bidentatae, Rhizoma Atractylodis, Pseudobulbus Cremastrae Seu Pleiones, Rhizoma Smilacis Glabrae, Rhizome Dioscoreae Septemlobae, Polistes mandarinus *Saussure*, Herba Lysimachiae, Sargassum, Radix Stephaniae Tetrandrae, Semen Coicis*

Jia 2010	*Cortex Phellodendri, Radix Achyranthis Bidentatae, Semen Coicis, Rhizoma Atractylodis, Radix Stephaniae Tetrandrae, Alisma orientalis, Radix Angelicae Pubescentis, Radix Saposhnikoviae, Pheretima*

Li and Zhang 2007	*Cortex Phellodendri, Radix Achyranthis Bidentatae, Semen Coicis, Rhizoma Atractylodis, Caulis Lonicerae, Rhizome Dioscoreae Septemlobae, Fructus Chaenomelis, Cortex Fraxini, Alisma orientalis, Radix Angelicae Sinensis*

Li and Song 2013	*Cortex Phellodendri, Radix Achyranthis Bidentatae, Semen Coicis, Rhizoma Atractylodis, Alisma orientalis, Pheretima, Rhizome Dioscoreae Septemlobae, Rhizoma Smilacis Glabrae, Semen Strychni*

Liu 2011	*Cortex Phellodendri, Radix Achyranthis Bidentatae, Semen Coicis, Rhizoma Atractylodis, Virgate Wormwood Herb, Rhizome Dioscoreae Septemlobae, Rhizoma Smilacis Glabrae, Wild Chrysanthemum, Radix Clematidis, Fructus Chaenomelis, Poria cocos, Radix Angelicae Pubescentis, Radix Glycyrrhizae*

Luo and Tang 2010	*Cortex Phellodendri, Radix Achyranthis Bidentatae, Semen Coicis, Rhizoma Atractylodis, Rhizome Dioscoreae Septemlobae, Plantain Seed, Herba Plantaginis, Caulis Lonicerae, Caulis Trachelospermi*

Mi 2016	*Rhizoma Smilacis Glabrae, Semen Coicis, Rhizome Dioscoreae Septemlobae, Spatholobus suberectus, Rhizoma Atractylodis, Cortex Phellodendri, Radix Achyranthis Bidentatae, Radix Polygoni Multiflori, Rhizoma Corydalis, Radix Clematidis, Alisma orientalis, Pheretima, Eupolyphaga Seu Steleophaga, Herba Leonuri, Virgate Wormwood Herb, Radix Glycyrrhizae*

Niu 2008	*Cortex Phellodendri, Radix Achyranthis Bidentatae, Semen Coicis, Rhizoma Atractylodis, Rhizoma Smilacis Glabrae, Rhizome Dioscoreae Septemlobae, Pheretima, Radix Stephaniae Tetrandrae, Fructus Chaenomelis, Flos Lonicerae*

Renbin et al. 2008	*Cortex Phellodendri, Radix Achyranthis Bidentatae, Semen Coicis, Rhizoma Atractylodis, Caulis Lonicerae, Rhizoma Smilacis Glabrae, Anemarrhena asphodeloides *Bunge*, Radix Paeoniae Rubra, Radix Clematidis, Rhizome Dioscoreae Septemlobae, Alisma orientalis, Zaocys Dhumnades*

Shi et al. 2008	*Cortex Phellodendri, Radix Achyranthis Bidentatae, Semen Coicis, Rhizoma Atractylodis, Caulis Lonicerae, Rhizoma Smilacis Glabrae, Radix Paeoniae Rubra, Gypsum, Anemarrhena asphodeloides *Bunge*, Ramulus Cinnamomi*

Sun 2006	*Cortex Phellodendri, Radix Achyranthis Bidentatae, Rhizoma Atractylodis, Rhizoma Smilacis Glabrae, Herba Siegesbeckiae, Radix Gentianae Macrophyllae, Semen Coicis, Herba Lysimachiae, Stigma Maydis, Cortex Erythrinae*

Tang et al. 2008	*Cortex Phellodendri, Radix Achyranthis Bidentatae, Semen Coicis, Rhizoma Atractylodis, Gypsum, Anemarrhena asphodeloides *Bunge*, Cortex Moutan, Radix Paeoniae Rubra, Radix Et Rhizoma Rhei, Alisma orientalis, Fructus Chaenomelis, Radix Clematidis*

Wang et al. 2009	*Cortex Phellodendri, Radix Achyranthis Bidentatae, Semen Coicis, Rhizoma Atractylodis, Rhizoma Smilacis Glabrae, Herba Plantaginis, Herba Lysimachiae, Caulis Lonicerae, Radix Et Rhizoma Rhei, Rhizome Dioscoreae Septemlobae, Rhizoma Polygoni Cuspidati, Radix Clematidis, Radix Salviae Miltiorrhizae, Radix Angelicae Sinensis*

Wang et al. 2016	*Cortex Phellodendri, Radix Achyranthis Bidentatae, Semen Coicis, Rhizoma Atractylodis, Ramulus Cinnamomi, Rhizoma Atractylodis Macrocephalae, Poria Cocos, Polyporus umbellatus, Alisma orientalis, Reed Rhizome, Lalang Grass Rhizome, Semen Persicae, Radix Glycyrrhizae*

T. Wang et al. 2016	*Ramulus Cinnamomi, Radix Cyathulae, Radix Paeoniae Rubra, Fructus Chaenomelis, Anemarrhena asphodeloides *Bunge*, Rhizoma Atractylodis, Cortex Phellodendri, Caulis Lonicerae, Rhizoma Smilacis Glabrae, Radix Clematidis, Gypsum, Semen Coicis, Herba Plantaginis, Herba Lysimachiae, Radix Glycyrrhizae*

Yu and Huang 2008	*Cortex Phellodendri, Radix Achyranthis Bidentatae, Rhizoma Atractylodis, Radix Gentianae Macrophyllae, Radix Paeoniae Rubra, Semen Coicis, Caulis Lonicerae, Rhizoma Smilacis Glabrae, Herba Taraxaci*

Zeng and Song 2013	*Cortex Phellodendri, Radix Achyranthis Bidentatae, Semen Coicis, Rhizoma Atractylodis, Rhizoma Smilacis Glabrae, Rhizome Dioscoreae Septemlobae, Radix Paeoniae Rubra, Caulis Lonicerae, Bombyx Batryticatus, Eupatorium adenophorum, Radix Glycyrrhizae*

Zhang and Chen 2012	*Cortex Phellodendri, Radix Achyranthis Bidentatae, Semen Coicis, Rhizoma Atractylodis, Caulis Lonicerae, Rhizoma Smilacis Glabrae, Rhizome Dioscoreae Septemlobae, Plantain Seed, Herba Lysimachiae, Pseudobulbus Cremastrae Seu Pleiones, Radix Gentianae Macrophyllae, Fructus Chaenomelis*

Zhao and Mai 2008	*Cortex Phellodendri, Radix Achyranthis Bidentatae, Semen Coicis, Rhizoma Atractylodis, Rhizoma Atractylodis Macrocephalae, Radix Glycyrrhizae*

Zhao 2014	*Cortex Phellodendri, Alisma orientalis, Radix Cyathulae, Radix Angelicae Sinensis, Rhizoma Atractylodis, Rhizome Dioscoreae Septemlobae, Fructus Chaenomelis, Cortex Fraxini, Semen Coicis, Caulis Lonicerae*

Zhu 2016	*Pheretima, Radix Saposhnikoviae, Radix Angelicae Pubescentis, Alisma orientalis, Radix Stephaniae Tetrandrae, Semen Coicis, Cortex Phellodendri, Radix Achyranthis Bidentatae, Rhizoma Atractylodis*

Note: MSD: modified Simiao decoction.
